# Supplementary material for: Intramuscular Polydeoxyribonucleotides in Fibrotic and Atrophic Localized Scleroderma: An Explorative Prospective Cohort Study
Source: Biomedicines. 2023 Apr 17;11(4):1190. doi: 10.3390/biomedicines11041190 (PMC10135559; doi:10.3390/biomedicines11041190)
Supplement: Supplementary file 1 [file biomedicines-11-01190-s001.zip › biomedicines-2286642-supplementary/Supplementay figures.pdf]

Supplementary Figures

Figure S1

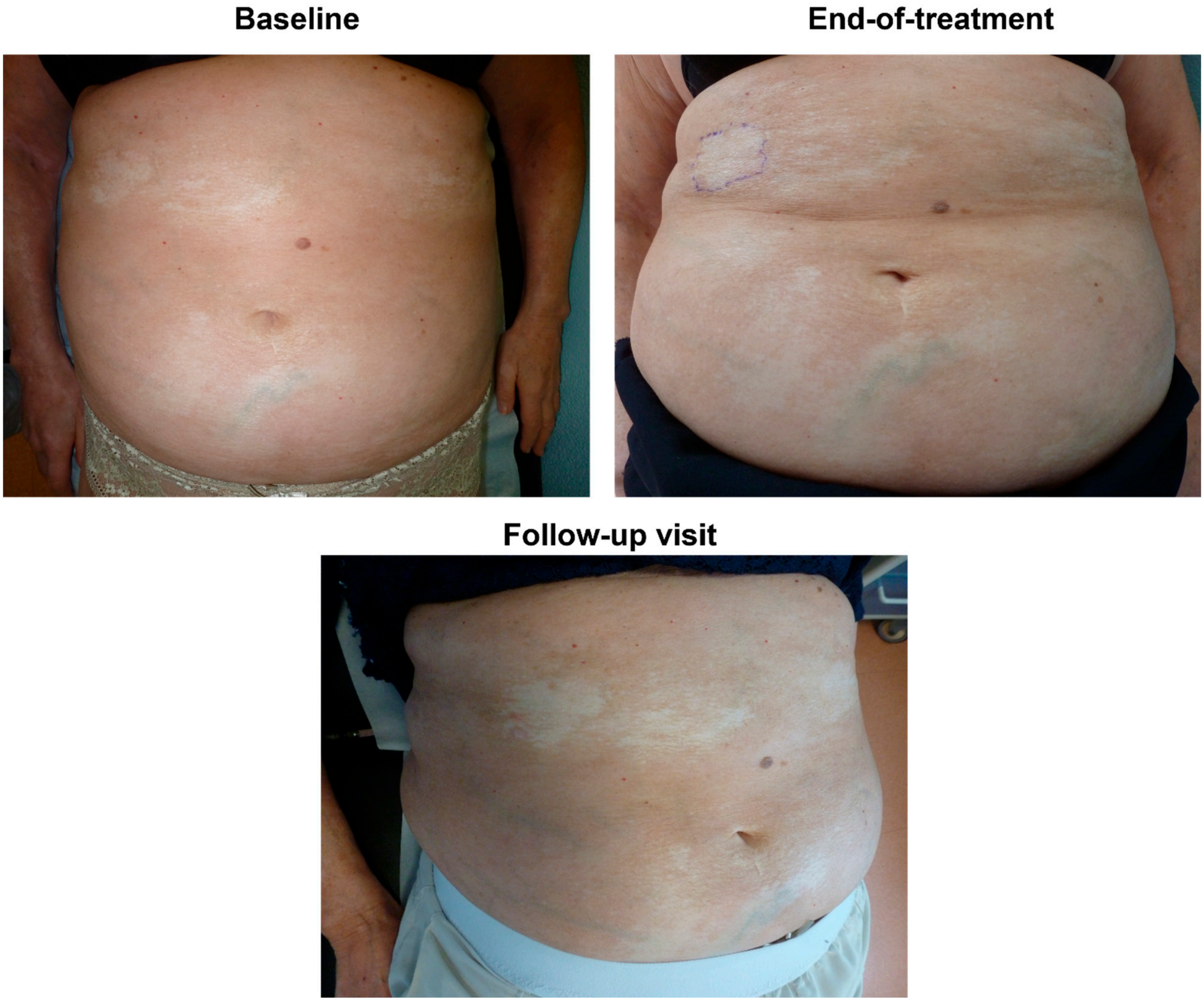

Figure S1: Clinical photograph of the progressive patient no. 3 showing the benefits of a three-month PDRN treatment after 90 and 180 days (“End-of-treatment and “Follow-up visit”, respectively) vs. baseline (Screening visit). Photographs owned by the authors with the patients’ permission of use.

Figure S2

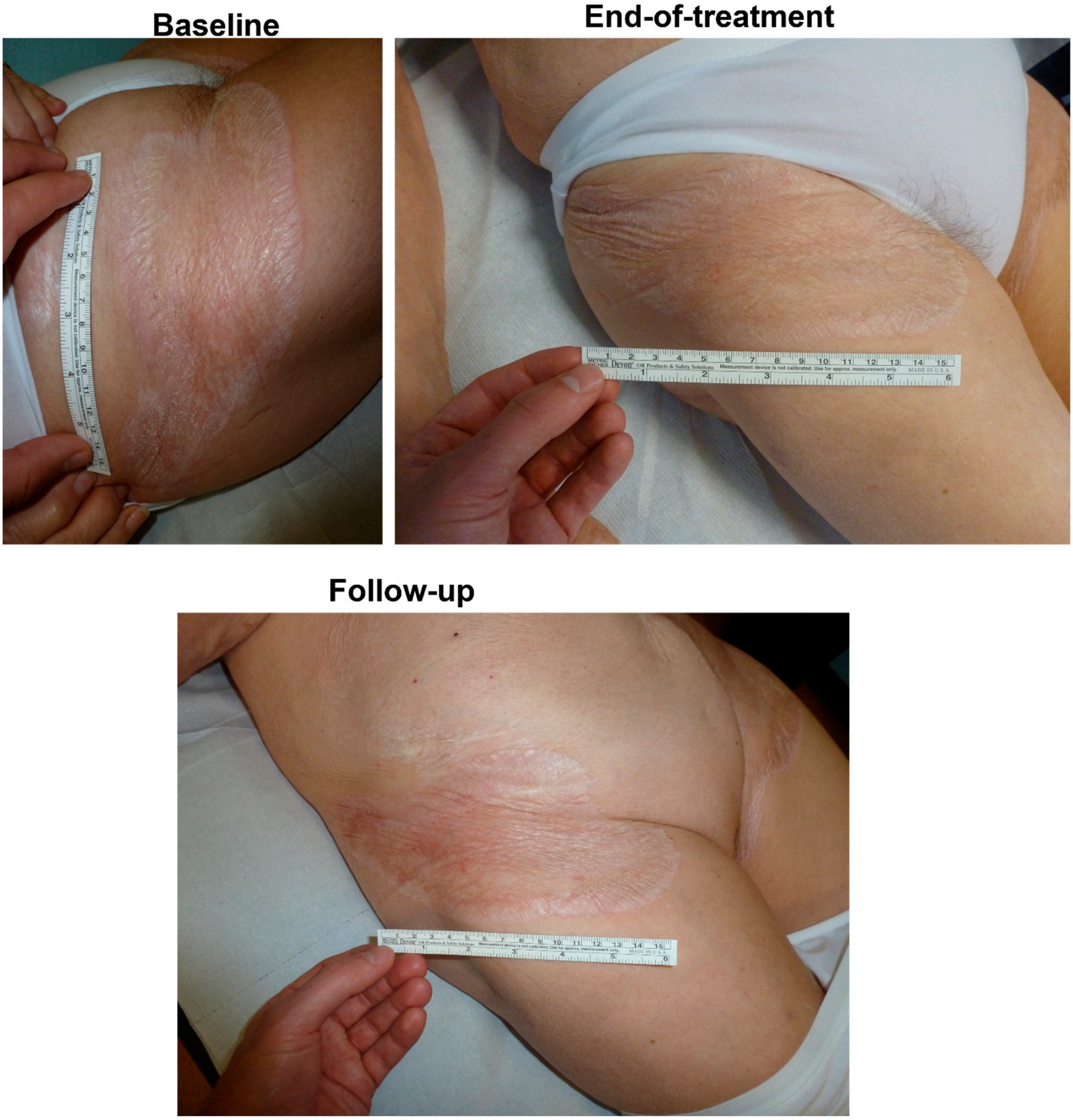

Figure S2: Clinical photograph of the progressive patient no. 4 showing the benefits of a three-month PDRN treatment after 90 and 180 days ("End-of-treatment" and "Follow-up" visit, respectively) vs. "baseline" (Screening visit). Photographs owned by the authors with the patients' permission of use.

Figure S3

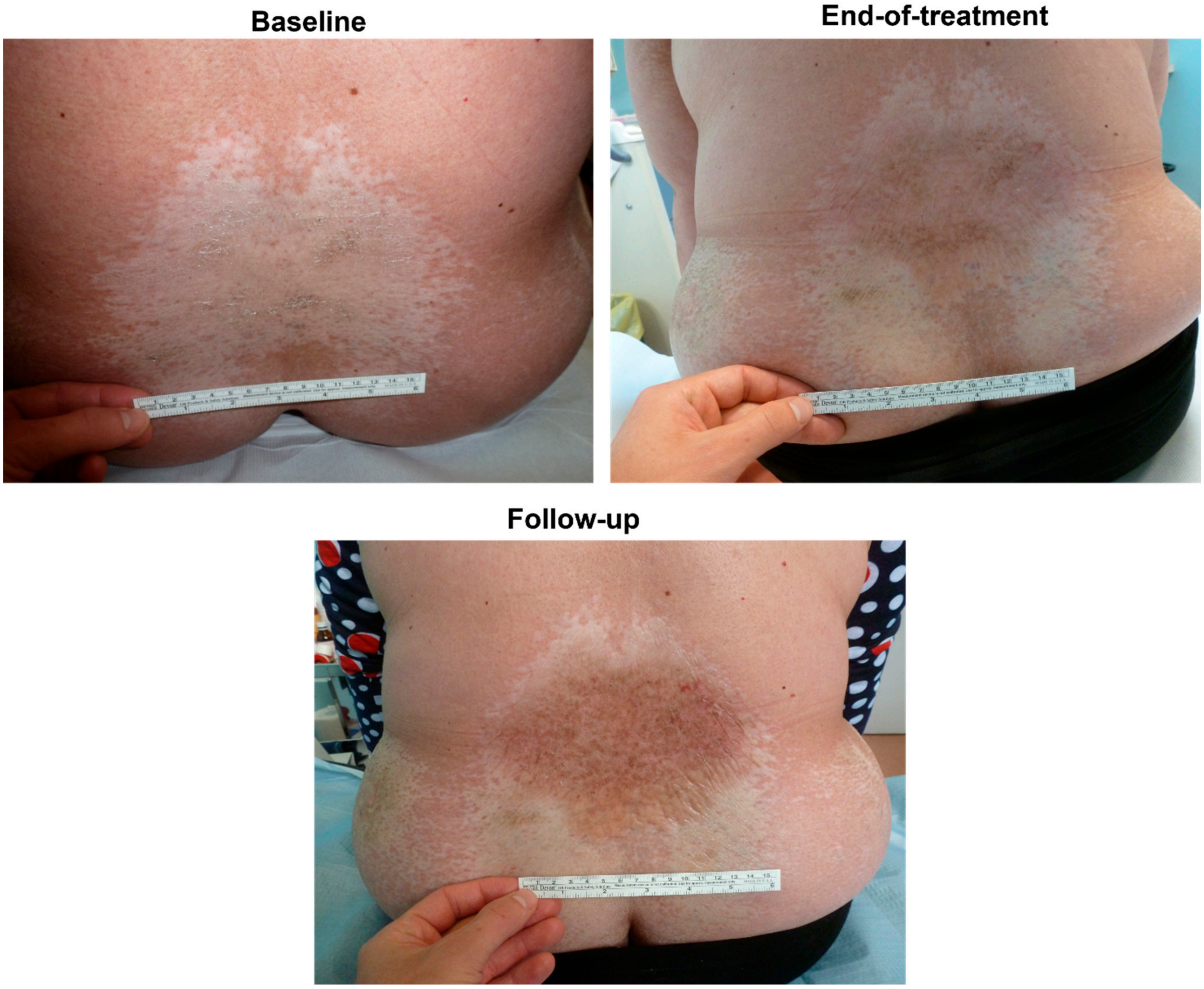

Figure S3: Clinical photograph of the progressive patient no. 14 showing the benefits of a three-month PDRN treatment after 90 and 180 days (“End-of-treatment” and “Follow-up” visit, respectively) vs. “baseline” (Screening visit). Photographs owned by the authors with the patients’ permission of use.

Figure S4

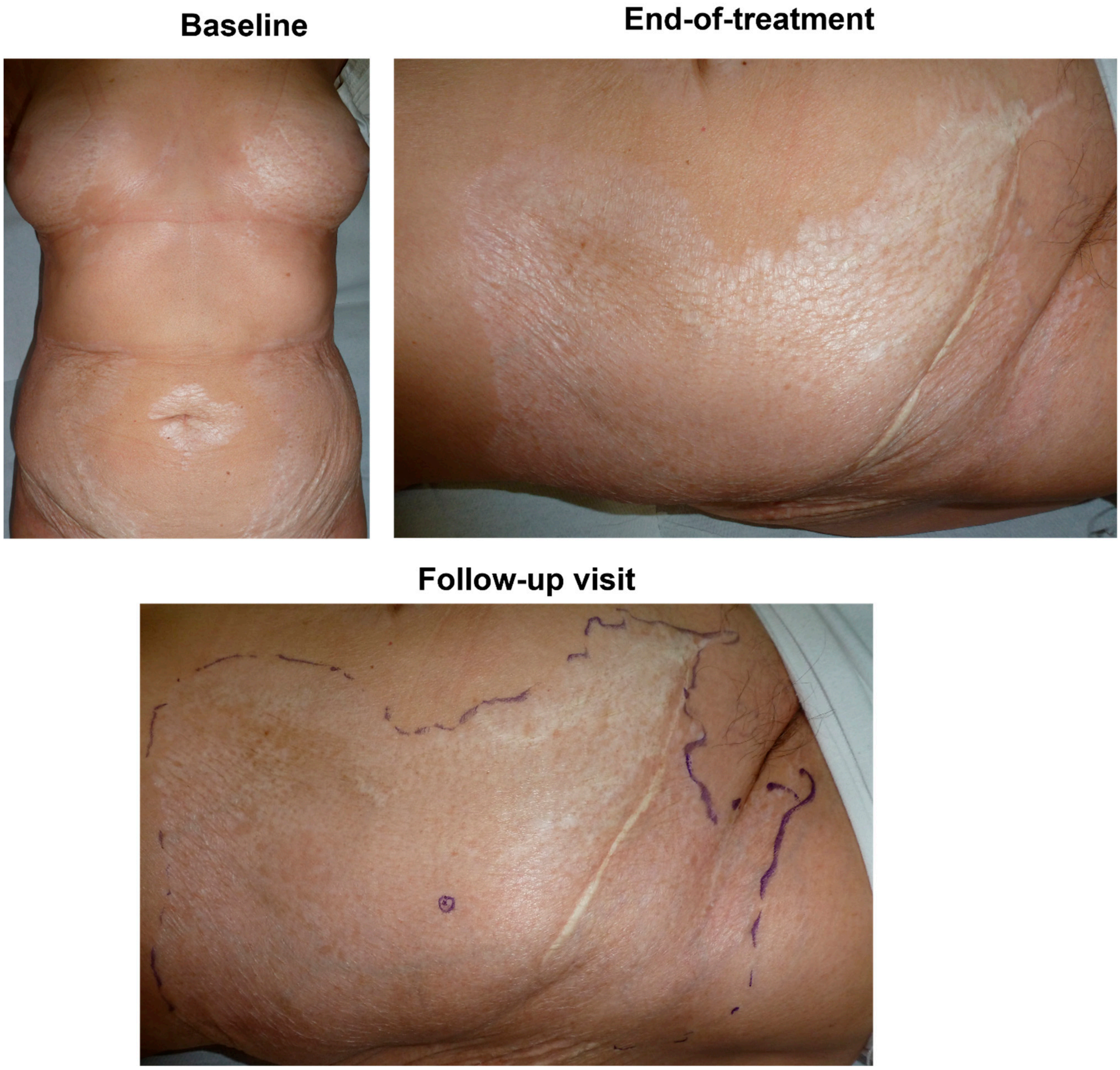

Figure S4: Clinical photograph of the progressive patient no. 16 showing the benefits of a three-month PDRN treatment after 90 and 180 days (“End-of-treatment” and “Follow-up” visit, respectively) vs. “baseline” (Screening visit). Photographs owned by the authors with the patients’ permission of use.
